# Body compositions phenotypes of older adults with COPD

**DOI:** 10.3389/fnut.2024.1449189

**Published:** 2024-10-07

**Authors:** Aleksandra Kaluźniak-Szymanowska, Dorota Talarska, Sławomir Tobis, Arkadiusz Styszyński, Szczepan Cofta, Katarzyna Wieczorowska-Tobis, Ewa Deskur-Śmielecka

**Affiliations:** ^1^Department of Palliative Medicine, Poznan University of Medical Sciences, Poznan, Poland; ^2^Department of Preventive Medicine, Poznan University of Medical Sciences, Poznan, Poland; ^3^Department of Occupational Therapy, Poznan University of Medical Sciences, Poznan, Poland; ^4^Department of Respiratory Medicine and Allergology, Poznan University of Medical Sciences, Poznan, Poland; ^5^Department of Human Nutrition and Dietetics, Poznan University of Life Sciences, Poznan, Poland

**Keywords:** body composition, phenotypes, COPD, older adults, sarcopenia, sarcopenic obesity, obesity

## Abstract

**Purpose:**

Changes in nutritional status are important extrapulmonary manifestations of the chronic obstructive pulmonary disease (COPD). The study aimed to assess the prevalence of different body composition phenotypes in older patients with COPD and to investigate the relationship between these phenotypes and the severity of the disease, as well as physical performance of the subjects.

**Patients and methods:**

The study included 124 subjects aged ≥60 with COPD. In all of them body composition analysis and muscle strength measurement were performed. Additionally, data from patients’ medical records were analyzed. Study sample was divided into four groups based on the phenotypic body composition: normal phenotype (N), sarcopenia, obesity and sarcopenic obesity (SO).

**Results:**

Incidence of sarcopenia was significantly higher in patients with severe or very severe COPD based on GOLD in comparison with subjects with mild or moderate obstruction (*p* = 0.043). Participants with sarcopenia, obesity and SO had lower results of the 6-min walk test than subjects with *N* (225.77 m, 275.33 m, 350.67 m, 403.56 m, respectively). Moreover, sarcopenia and SO had lower results than obesity (*p* = 0.001, *p* = 0.041, respectively).

**Conclusion:**

Sarcopenia is common in patients with advanced COPD. Sarcopenia and SO are associated with poorer physical performance. All older people with COPD should routinely have their body composition assessed, instead of simply measuring of body weight or body mass index (BMI).

## 1 Introduction

According to the Global Initiative for Chronic Obstructive Lung Disease (GOLD) from 2023, chronic obstructive pulmonary disease (COPD) is one of the top three leading causes of death worldwide (1). Estimates suggest that the prevalence of diagnosed COPD among older individuals (21%) is almost twice as high as in the general population (12%) (2).

Changes in nutritional status are essential extrapulmonary manifestations of COPD. These include obesity and sarcopenia, which coexist with a chronic inflammatory state and negatively affect the course of the disease.

The rate of catabolic processes in COPD patients is increased, directly leading to muscle protein damage. Additionally, the synthesis of muscle proteins is disrupted in these subjects (3, 4). On the other hand, COPD, especially during exacerbations, results in reduced physical activity and decreased food intake (including protein-rich foods), which may lead to both muscle mass and strength loss (4). While sarcopenia is common among patients with COPD, its prevalence varies depending on the diagnostic criteria used. When a diagnosis of sarcopenia is based solely on low muscle mass, its prevalence in COPD patients is as high as 34%. Combining low muscle mass with low muscle strength or physical performance results in an essentially lower percentage (15.5%) of diagnosed sarcopenia (3). Regardless of the diagnostic criteria, sarcopenia is significantly more frequent in patients with COPD stage 3 or 4 and is associated with decreased quality of life, more frequent hospitalizations, and increased risk of death (3–7).

Obesity is most often observed in patients with mild to moderate obstruction (16–24%) and less frequently in those with severe obstruction (6%) (8). Excessive body weight in a subject with COPD may result in decreased exercise capacity and exacerbation of dyspnea, negatively affecting the patient’s quality of life (9). Even though obesity is a risk factor for numerous metabolic diseases, an “obesity paradox” was described in subjects with COPD: the lowest mortality was observed in individuals with a Body Mass Index (BMI) above the norm, i.e., >25 kg/m2 (but less than 32 kg/m2). The mechanism behind this phenomenon remains unclear. Presumably, it is the high muscle mass in obese individuals that contributes to their better prognosis rather than the high total body mass (8, 10, 11). Another explanation of the “obesity paradox” may be that nutritional status is assessed with BMI, which is not a measure of body composition (12). It should be emphasized that obesity, understood as a high percentage of body fat, may coexist with low muscle mass (especially in older people). This condition is termed sarcopenic obesity. This phenomenon is supposed to be more common in COPD patients than in the general population (12) and is associated with a worse prognosis than obesity or sarcopenia independently (12–16).

Studies comparing body composition phenotypes in COPD patients are sparse (14, 15), and none uses current diagnostic criteria for sarcopenia and sarcopenic obesity. Therefore, we assessed the frequency of different body composition phenotypes in older individuals with COPD and evaluated the relationship between these phenotypes, the disease severity, and the subjects’ physical performance.

## 2 Materials and methods

Our cross-sectional study was conducted on patients from the Pulmonary Rehabilitation Ward (Great Poland Centre of Pulmonology and Thoracic Surgery) from September 2019 to November 2020. The inclusion criteria were: diagnosis of COPD based on GOLD criteria (1), age of 60 years or older, cognitive performance enabling the interview, and written consent to participate in the study. The exclusion criteria included contraindications for body composition analysis with the bioimpedance method (e.g., pacemaker, metallic elements in the body) and active cancer.

Each subject gave written informed consent before the study, conducted under the Declaration of Helsinki. The study protocol was approved by the Bioethical Committee of the Poznan University of Medical Sciences, Poland (approval No: 888/19).

All participants had cognitive function assessment, body composition analysis with the electrical bioimpedance method, and muscle strength measurements. Additional data were extracted from the hospital database, including results of the spirometry, the 6-min walk test (6MWT), and CRP levels as a marker of inflammatory status.

### 2.1 Cognitive function assessment

We used the Abbreviated Mental Test Score (AMTS) to assess participants’ mental capacity (17). The questionnaire contains 10 items scored 1 point each. Subjects scoring <7 points were excluded from further investigation due to suspected significant cognitive impairment.

### 2.2 Body composition analysis

The electrical bioimpedance method with the InBody 120 analyzer (Biospace, Seoul, South Korea) was used to assesses subjects’ body composition. Segmental impedance measurements (right arm, left arm, trunk, right leg, left leg) were performed after at least a 2-h fasting period using eight self-adhesive electrodes. The following body composition parameters were analyzed: total body weight, BMI, total body water (TBW), percentage of body fat (PBF), skeletal muscle mass (SMM), and fat-free mass (FFM).

### 2.3 Sarcopenia

We applied the European Working Group on Sarcopenia in Older People 2 (EWGSOP2) criteria for the diagnostics of sarcopenia (18). As COPD raises clinical suspicion of sarcopenia, muscle strength and muscle mass were assessed in all subjects.

Upper limb strength was evaluated using a hand dynamometer (Saehan, Changwon, Korea). Measurements were taken in a seated position, with subjects holding the elbow at 90° of flexion. Each arm was tested twice. A better result was compared to the cut-off points proposed by EWGSOP2. Low muscle strength was defined as less than 16 kg for women and less than 27 kg for men (18).

Lower limb strength was assessed based on the results of the five-repetition sit-to-stand test. Participants were instructed to cross their arms over their chests and to stand up and sit down five times (unassisted) as quickly as possible. The time of more than 15 s indicated a low lower limb strength. Low upper or lower limb strength was a premise of sarcopenia.

To confirm the diagnosis of sarcopenia, muscle mass was assessed based on the electrical bioimpedance analysis results. Following the EWGSOP2 algorithm, we used the Appendicular Lean Mass (ALM) index, which is the sum of the fat-free mass of the upper and lower limbs (kg) divided by height in meters squared (m2). Low muscle mass was defined using the cut-off points developed for the Polish population: <5.6 kg/m^2^ for women and < 7.4 kg/m^2^ for men (19). Sarcopenia was diagnosed in subjects with concomitant low muscle mass and strength.

### 2.4 Obesity

The percentage of body fat from electrical bioimpedance analysis was a measure of obesity. The cut-off points for defining excessive body fat were > 42% for women and > 30% for men (20–22).

### 2.5 Sarcopenic obesity

Modified guidelines from the European Society for Clinical Nutrition and Metabolism (ESPEN) and the European Association for the Study of Obesity group experts (EASO) were used to diagnose sarcopenic obesity (23). The modification consisted of evaluating muscle mass with the ALM/BMI index instead of the ALM/body mass. Such modification is commonly used in diagnosing sarcopenic obesity (24, 25). The ALM/BMI index cut-off points were < 0.512 for women and < 0.789 for men (26).

Sarcopenic obesity was diagnosed in subjects with concomitant high BMI (≥ 30 kg/m2), reduced muscle strength (as described above), increased fat mass (as described above) and reduced muscle mass (based on ALM/BMI index).

The study sample was divided based on the body composition phenotypes into groups: normal phenotype, sarcopenia, obesity, and sarcopenic obesity (SO).

### 2.6 Statistical analysis

Qualitative variables are presented as numbers (*n*) and percentages (%). Quantitative data are described by means and standard deviations. Differences between the four phenotype groups were assessed using:

- Analysis of Variance (ANOVA).
- ANOVA with *F* correction for body mass, BMI, BFM, PBF, ALM/BMI index, upper and lower limb muscle strength.
- Kruskal-Wallis ANOVA for number of comorbidities and CRP levels.

Differences between groups were evaluated using post-hoc tests. The frequency of specific body composition phenotypes in two groups was compared using Pearson’s Chi-squared test with Yates’ correction. A *p*-value <0.05 was considered significant. All statistical analyses were performed in the PQStat program (PQStat Software, Poland).

## 3 Results

### 3.1 Characteristics of the study sample

**Figure 1:**
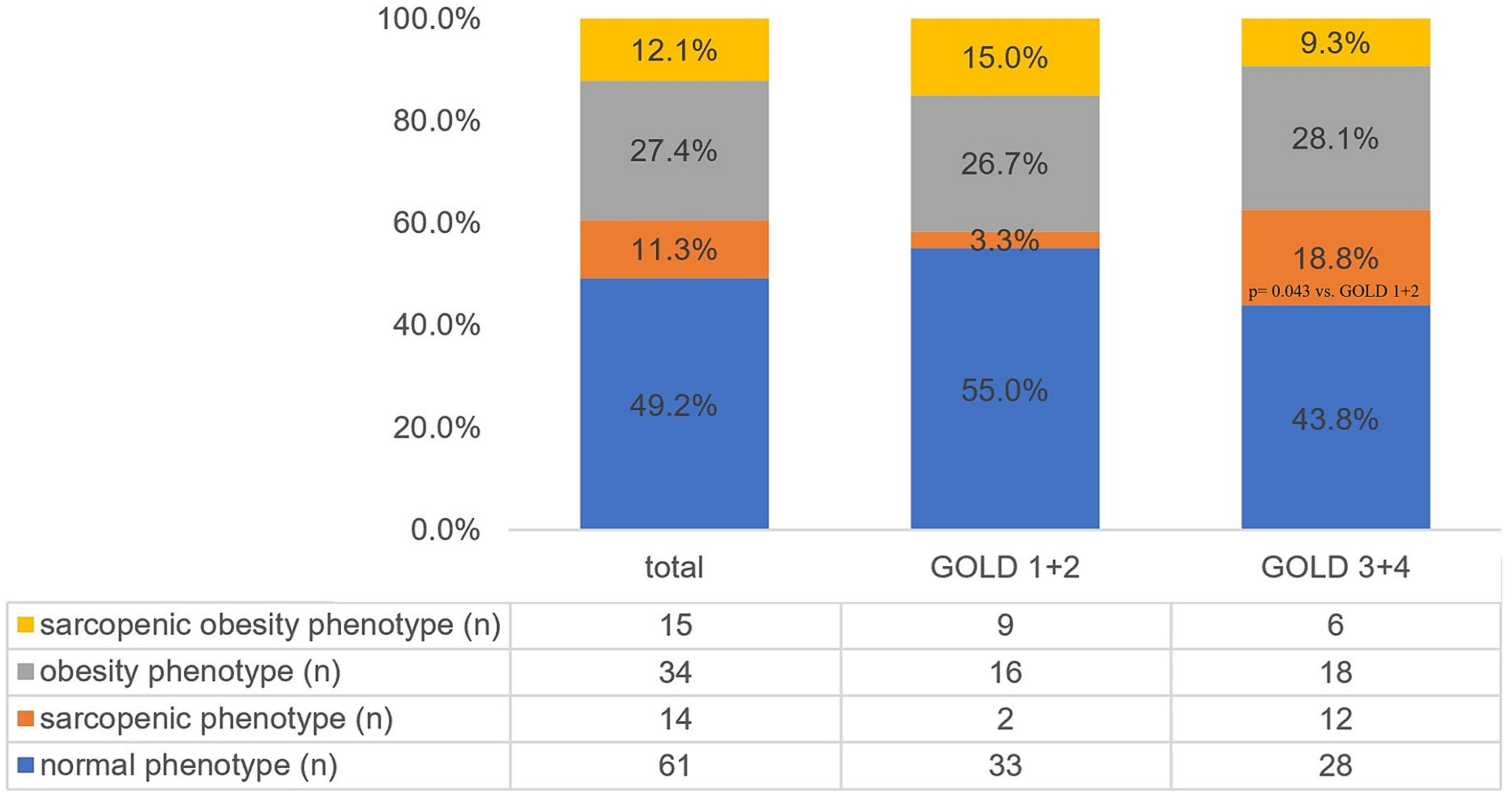
Prevalence of different body composition phenotypes in the total study sample and stratified by COPD severity. GOLD, Global Initiative for Chronic Obstructive Lung Disease.

The study involved 124 individuals (40.3% women) with COPD. The mean age was 69.4 ± 6.1 years. COPD stage 1 was diagnosed in 9 individuals, stage 2—in 51 individuals, stage 3—in 52 individuals, and stage 4—in 12 individuals. Due to the small sample sizes of patients with COPD stages 1 and 4, further analysis combined stages 1 and 2 (GOLD 1 + 2) and stages 3 and 4 (GOLD 3 + 4). Figure 1 presents the frequency of body composition phenotypes for the entire study sample and stratified by COPD severity. Sarcopenia was more frequent in patients with stages 3 and 4 compared to individuals with COPD stage 1 + 2 (*p* = 0.043).

### 3.2 Anthropometric characteristics

Table 1 presents the anthropometric parameters of the subgroups with different body composition phenotypes. Individuals with sarcopenia had significantly lower body weight, BMI, body water content, fat tissue content, skeletal muscle mass, limb lean mass, and ALM index than other study participants. Patients with sarcopenic obesity had the lowest ALM/BMI ratio. The handgrip test results were significantly lower in individuals with sarcopenia than those with normal phenotype or obesity (*p* < 0.001 and *p* < 0.001, respectively). Subjects with SO had significantly lower upper limb strength compared to patients with obesity (*p* = 0.004). The results of the five-repetition sit-to-stand test did not differ between the body composition phenotype groups.

**Table 1:** Anthropometric characteristics of the study sample divided by body composition phenotype.

	Normal phenotype (1)	Sarcopenic phenotype (2)	Obesity phenotype (3)	Sarcopenic obesity phenotype (4)	*p*-value
Age (years)	69.6 ± 6.3 (69.0; 65.0–74.0)	69.2 ± 7.0 (68.0; 64.0–72.3)	68.8 ± 5.1 (68.5; 65.3–71.8)	69.7 ± 6.6 (69.0; 66.5–71.0)	0.909
Sex – *n* (%)					
Males	31 (50.8%)	8 (57.1%)	25 (73.5%)	10 (66.7%)	0.169
Females	30 (49.2%)	6 (42.9%)	9 (26.5%)	5 (33.3%)	
Height (cm)	166.6 ± 9.5 (165.0; 161.0–175.0)	162.5 ± 8.7 (161.5; 159.3–169.8)	169.1 ± 8.8 (170.0; 164.0–175.0)	163.5 ± 8.7 (167.0; 156.5–169.5)	0.076
Body mass (kg)	70.2 ± 13.1 (71.1; 62.5–77.5)	52.6 ± 9.1 (52.1; 47.0–56.9)^***^	93.5 ± 15.0 (93.0; 83.8–98.0)^***, ###^	98.2 ± 21.0 (96.1; 82.8–110.6)^***, ###^	0.000
BMI (kg/m2)	25.2 ± 3.7 (25.1; 22.9–27.2)	19.9 ± 3.2 (19.7; 18.0–20.4)^***^	32.7 ± 4.6 (32.5; 30.0–34.9)^***, ###^	36.5 ± 6.0 (34.8; 32.7–41.8)^***, ###^	0.000
TBW (kg)	37.2 ± 7.6 (37.7; 30.8–42.3)	29.4 ± 4.9 (28.3; 25.5–33-3)^**^	42.1 ± 7.5 (42.1; 36.0–47.2)^**, ###^	41.1 ± 9.0 (40.7; 35.7–46.4)^###^	0.000
BFM (kg)	19.6 ± 7.5 (18.9; 14.8–24.5)	12.7 ± 4.5 (13.0; 9.5–15.8)^**^	36.3 ± 8.3 (35.3; 30.2–40.2)^***, ###^	42.7 ± 10.4 (38.3; 34.8–52.4)^**, ###^	<0.001
SMM (kg)	27.8 ± 6.2 (28.0; 22.4–32.0)	21.4 ± 4.0 (20.4; 18.2–24.4)^**^	31.7 ± 6.1 (31.4; 27.0–35.8)^**, ###^	30.7 ± 7.2 (30.9; 26.4–34.9)^###^	0.000
PBF (%)	27.6 ± 8.4 (27.3; 20.2–35.9)	23.6 ± 7.5 (24.6; 18.6–27.8)	38.8 ± 6.0 (36.9; 33.5–44.4)^***, ###^	43.4 ± 4.0 (44.5; 40.6–45.8)^***, ###, $^	<0.001
ALM (kg)	20.4 ± 5.1 (21.5; 16.2–24.2)	15.4 ± 3.4 (14.3; 12.5–18.1)^**^	24.0 ± 4.8 (24.4; 20.0–27.4)^**, ###^	23.1 ± 5.9 (22.8; 19.5–26.2)^###^	<0.001
ALM index (kg/m2)	7.3 ± 1.2 (7.3; 6.5–8.1)	5.8 ± 0.8 (5.6; 5.2–6.2)^***^	8.3 ± 1.1 (8.2; 7.5–9.1)^***, ###^	8.5 ± 1.6 (8.7; 7.2–9.7)^***, ###^	<0.001
Low muscle mass – *n* (%)	12 (19.7%)	14 (100%)^***^	1 (2.9%)^*, ###^	1 (6.7%)^###^	<0.001
ALM/BMI index	0.82 ± 0.19 (0.83; 0.65–0.98)	0.78 ± 0.16 (0.82; 0.68–0.88)	0.74 ± 0.15 (0.79; 0.64–0.83)	0.63 ± 0.11 (0.67; 0.49–0.72)^***, #, $^	<0.001
Upper limb strength (kg)	28.7 ± 9.7 (25.1; 21.8–38.0)	19.5 ± 4.9 (19.1; 16.2–24.0)^***^	32.8 ± 8.1 (32.3; 27.6–36.0)^###^	22.0 ± 9.1 (21.5; 14.0–30.5)^$$^	<0.001
Low upper limb strength – *n* (%)	16 (26.4%)	12 (85.7%)^***^	5 (14.7%)^###^	13 (86.7%)^***, $$$^	<0.001
Lower limb strength (s)	13.2 ± 4.2 (12.7; 10.1–14.9)	16.6 ± 6.5 (16.9; 11.3–18.6)	14.4 ± 6.2 (12.4; 10.4–15.7)	20.6 ± 9.5 (17.1; 14.3–24.6)	0.022
Low lower limb strength – *n* (%)	16 (26.4%)	8 (57.1%)^*^	8 (23.5%)^#^	10 (66.7%)^*, $^	0.003

Values are presented as numbers (%) or mean ± standard deviation (median; interquartile range) for descriptive analyses.

^*^p < 0.05 vs 1; ^**^p < 0.01 vs 1; ^***^p < 0.001 vs 1.

^#p < 0.05 vs 2; ###p < 0.001 vs 2.^

^$^*p* < 0.05 vs 3; ^$$^*p* < 0.01 vs 3; ^$$$^*p* < 0.001 vs 3.

BMI, body mass index; TBW, total body water; BFM, body fat mass; SMM, skeletal muscle mass; PBF, percent body fat; ALM, appendicular lean mass.

### 3.3 Clinical characteristics

Table 2 presents the clinical characteristics of the study subjects divided by body composition phenotypes. Although no differences were observed in the number of comorbidities between the body composition phenotype groups, individuals with obesity or SO had more prescription medications than those with a normal phenotype (*p* = 0.031 for both comparisons). Participants with sarcopenia had significantly lower Tiffeneau index compared to those with obesity and SO (*p* = 0.039 and *p* = 0.003, respectively). They also had lower FEV1 than subjects with normal phenotype and those with SO (*p* = 0.007 and *p* = 0.025, respectively).

**Table 2:** Clinical characteristics of the study sample divided by body composition phenotypes.

	Normal phenotype (1)	Sarcopenic phenotype (2)	Obesity phenotype (3)	Sarcopenic obesity phenotype (4)	*p*-value
Comorbidities (number)	3.0 ± 1.1 (3.0; 2.0–4.0)	2.8 ± 1.1 (3.0; 2.0–3.0)	3.3 ± 1.2 (3.0; 3.0–4.0)	3.8 ± 1.4 (3.0; 3.0–4.5)	0.078
Drugs (number)	7.2 ± 4.1 (7.0; 4.0–10.0)	7.7 ± 4.0 (6.0; 5.0–11.8)	9.7 ± 4.3 (10.0; 6.3–12.0)^*^	10.0 ± 3.3 (10.0; 8.0–12.5)^*^	0.009
6 MWT (m)	403.6 ± 111.0 (420.0; 360.0–472.5)	225.8 ± 124.6 (210.0; 130.0–375.0)^**^	350.7 ± 125.0 (392.5; 332.5–427.5)^*, ##^	275.3 ± 100. (240.0; 202.5–375.0)^**, $^	<0.001
FEV1	53.2 ± 19.0 (52.0; 38.0–66.0)	38.3 ± 15.1 (36.5; 30.8–43.5)^**^	49.9 ± 17.8 (45.5; 38.5–61.8)	53.8 ± 20.6 (51.0; 39.0–61.5)^#^	0.053
FEV1/FVC	50.7 ± 11.6 (52.7; 41.5–60.3)	43.6 ± 9.3 (42.0; 38.1–52.0)	51.7 ± 14.2 (53.2; 44.5–62.9)^#^	57.5 ± 13.2 (62.3; 47.9–69.4)^##^	0.028
CRP	5.8 ± 7.5 (2.2; 0.8–7.6)	3.2 ± 3.5 (2.3; 1.1–2.8)	5.2 ± 5.1 (3.1; 1.5–9.1)	4.7 ± 3.2 (4.2; 2.1–7.7)	0.519

Values are presented as numbers (%) or mean ± standard deviation (median; interquartile range) for descriptive analyses.

^*^p < 0.05 vs 1; ^**^p < 0.01 vs 1.

^#p < 0.05 vs 2; ##p < 0.01 vs 2.^

^$^*p* < 0.05 vs 3.

6MWT, 6 min walk test; FEV1/FVC, forced expiratory volume in 1 s/forced vital capacity; FEV1, forced expiratory volume in 1 s; CRP, C-Reactive Protein.

The most distinct differences were noted in the 6MWT results. All groups with abnormal body composition phenotypes had lower 6MWT results than subjects with N. Individuals with sarcopenia and SO had worse results than those in the obesity group (*p* = 0.001 and *p* = 0.041, respectively).

The levels of the inflammation markers did not differ between the compared groups.

## 4 Discussion

We assessed the prevalence of various body composition phenotypes in older patients with COPD and described their clinical characteristics. Pathological body composition phenotypes were common in our study sample, with obesity affecting 27.4% of subjects (*n* = 34), followed by SO in 12.1% (*n* = 16) and sarcopenia in 11.3% (*n* = 14). To the best of our knowledge, few studies have focused on the assessment of body composition phenotypes in individuals with COPD and their association with disease severity (14, 15); none of them have utilized the up-to-date diagnostic criteria [EWGSOP2 for sarcopenia (18), ESPEN and EASO for sarcopenic obesity (23)]. Therefore, our results contribute to the topic.

In our previous study, including 211 community-dwelling individuals aged 60 and over in Poland, the prevalence of obesity was 32.7%, SO 7.1%, and sarcopenia 10% (27). Both SO and sarcopenia are more common in the current study sample. The reason for the higher prevalence of these pathological phenotypes in COPD patients remains unclear. However, factors that may contribute to muscle mass and strength loss, such as reduced physical activity, elevated levels of pro-inflammatory cytokines with concurrent oxidative stress, and reduced energy intake were described in COPD patients (5, 28, 29). Moreover, COPD exacerbations are associated with rapid protein degradation and often immobilization of patients, aggravating muscle mass loss. Corticosteroids used to treat COPD exacerbations may further enhance muscle protein catabolism (8, 10).

So far, only two studies assessed the distribution of all four body composition phenotypes in patients with COPD (14, 15). Joppa et al., (15) in a multicenter prospective study (ECLIPSE) involving 2000 individuals with COPD (mean age 63.5 ± 7.1), showed that sarcopenia was present in 24.2%, obesity in 15.4%, and SO in 9.8% of subjects (15). Machado et al. (14) performed a retrospective analysis of body composition phenotypes in 270 patients with COPD recruited for a physical training program. Sarcopenia was diagnosed in 21%, obesity in 13%, and SO in as many as 27% of participants (14). The higher prevalence of sarcopenia in these studies compared to our findings can be explained by the difference in the diagnostic criteria. The authors of the previous studies diagnosed sarcopenia based on low muscle mass and excluding low muscle strength criterion, currently recognized as the most critical component of sarcopenia (18). Including this second criterion in our analysis presumably resulted in a lower prevalence of the condition. The omission of the low muscle strength criterion may also explain the higher prevalence of SO in the study by Machado et al. Although Joppa et al. also ignored muscle strength in the diagnostics of sarcopenic obesity, their results were similar to our findings. It should be noted, however, that their study was not limited to older individuals. Including younger adults, in whom SO is less common than in the older population, may explain the discrepancy between the results of Joppa et al. and Machado et al. and the apparent similarity to our findings.

The severity of COPD did not differ between individuals with obesity or SO compared to those with normal phenotype. However, subjects with sarcopenia more often had severe or very severe obstruction (GOLD 3 + 4). These findings align with a systematic review and meta-analysis conducted by Sepúlveda-Loyola et al. (3). The analysis, which included 23 studies with 9,637 participants aged ≥40, showed that sarcopenia was present in 37.6% of individuals with COPD stage 3 or 4 compared to 19.1% of subjects with stage 1 or 2 (*p* = 0.020) (3).

We observed worse results of the 6MWT in individuals with sarcopenia and SO compared to those with normal and obesity phenotypes. Similar findings were reported in the previously discussed studies (14, 15). In our study the mean distance in the 6MWT was 275.33 m (± 99.97 m) in subjects with SO and only 225.77 m (± 124.55 m) in those with sarcopenia. The 6MWT is widely used to assess exercise tolerance. Although there are no cut-off points for this test, it has been documented that a distance of less than 350 m is associated with a worse prognosis and increased risk of death in COPD patients (30, 31). The more severe course of COPD we observed in patients with sarcopenic obesity poses a challenge in relation to the so-called obesity paradox. We believe that the better clinical outcomes of COPD patients with obesity presented in numerous studies do not apply to people with SO, which is an argument for taking body composition phenotypes into account when determining the prognosis in severe forms of COPD. As physical performance is largely dependent on COPD severity, it should have been interesting to investigate differences in functional parameters in patients with abnormal body phenotypes divided according to the obstruction severity. Such analysis could contribute to the explanation of the obesity paradox.

Although we do not know the direction of causality of our results, they have important clinical implications. It seems reasonable to perform routine assessments of nutritional status, especially in people with severe or very severe obstruction, who are more likely to have abnormal body composition phenotypes and, at the same time, show a worse clinical prognosis.

A strong point of our analysis is that, to the best of our knowledge, it is the first study evaluating the prevalence of various body composition phenotypes in older individuals with COPD based on the up-to-date diagnostic criteria (EWGSOP2, ESPEN, EAOS) and investigating association between body composition phenotype and patient’s clinical status.

Our study has some limitations. First of all, we used bioimpedance methods instead of DEXA which according to experts (23) may lead to underestimation of fat mass while overestimating lean body mass. This may result in failure to recognize sarcopenic obesity in people with high BMI. Secondly, the study group consisted exclusively of individuals participating in a pulmonary rehabilitation program whose general health condition was good enough for such a modality. Therefore, our results cannot be generalized to the broader population of individuals with COPD. Furthermore, our study lacks information on smoking, which may affect both the severity of the disease and the nutritional status of the participants. Additionally, due to the cross-sectional nature of our study, no causal inferences can be made.

In conclusion, pathological body composition phenotypes involve more than half of the COPD older patients, which is visibly higher than in the general elderly population. Sarcopenia and sarcopenic obesity are associated with reduced physical performance, and sarcopenia is more prevalent in subjects with advanced stages of the disease. Based on our results, body composition assessment in addition to body mass measurement and BMI calculation contributes to the picture and should be performed in all older people with COPD.

## Data Availability

All relevant data are within the manuscript and are openly available in the Zenodo repository: https://doi.org/10.5281/zenodo.10039309.
